# I-123 iomazenil single photon emission computed tomography for detecting loss of neuronal integrity in patients with traumatic brain injury

**DOI:** 10.1186/s13550-017-0276-1

**Published:** 2017-03-23

**Authors:** Kagari Abiko, Katsunori Ikoma, Tohru Shiga, Chietsugu Katoh, Kenji Hirata, Yuji Kuge, Kentaro Kobayashi, Nagara Tamaki

**Affiliations:** 1 Department of Rehabilitation Medicine, Hokkaido University Hospital, Sapporo, 060-8638 Japan; 2 Department of Nuclear Medicine, Hokkaido University School of Medicine, North 15th, West 7th, Kitaku, Sapporo, 060-8638 Japan; 3 Department of Tracer Kinetics, Hokkaido University, Sapporo, 060-8638 Japan

## Abstract

**Background:**

Traumatic brain injury (TBI) causes brain dysfunction in many patients. Using C-11 flumazenil (FMZ) positron emission tomography (PET), we have detected and reported the loss of neuronal integrity, leading to brain dysfunction in TBI patients. Similarly to FMZ PET, I-123 iomazenil (IMZ) single photon emission computed tomography (SPECT) is widely used to determine the distribution of the benzodiazepine receptor (BZR) in the brain cortex. The purpose of this study is to examine whether IMZ SPECT is as useful as FMZ PET for evaluating the loss of neuronal integrity in TBI patients.

The subjects of this study were seven patients who suffered from neurobehavioral disability. They underwent IMZ SPECT and FMZ PET. Nondisplaceable binding potential (BP_ND_) was calculated from FMZ PET images. The uptake of IMZ was evaluated on the basis of lesion-to-pons ratio (LPR). The locations of low uptake levels were visually evaluated both in IMZ SPECT and FMZ PET images. We compared FMZ BP_ND_ and (LPR-1) of IMZ SPECT.

**Results:**

In the visual assessment, FMZ BP_ND_ decreased in 11 regions. In IMZ SPECT, low uptake levels were observed in eight of the 11 regions. The rate of concordance between FMZ PET and IMZ SPECT was 72.7%. The mean values IMZ (LPR-1) (1.95 ± 1.01) was significantly lower than that of FMZ BP_ND_ (2.95 ± 0.80 mL/mL). There was good correlation between FMZ BP_ND_ and IMZ (LPR-1) (*r* = 0.80).

**Conclusions:**

IMZ SPECT findings were almost the same as FMZ PET findings in TBI patients. The results indicated that IMZ SPECT is useful for evaluating the loss of neuronal integrity. Because IMZ SPECT can be performed in various facilities, IMZ SPECT may become widely adopted for evaluating the loss of neuronal integrity.

## Background

Traumatic brain injury (TBI) causes brain dysfunction in many patients. Patients with head injury who do not show residual focal neurological symptoms, such as paralysis and aphasia, are often discharged to return home after the acute phase, without confirmation of the need for rehabilitation. However, even patients who had suffered a mild head injury may experience after-effects including fatigue, memory disorder, and poor concentration, which significantly and adversely affect their daily life [1–3]. Neurobehavioral disability such as impaired attention, memory disorder, and executive function disorder will interfere with patients’ education and employment opportunities, causing serious economic losses and significant decrease in the quality of their daily life. Therefore, it is important to promptly make an accurate diagnosis, understand the clinical conditions, and provide rehabilitation and support to patients on the basis of such a diagnosis.

Because many of neurobehavioral disability caused by TBI cannot be explained only by the site of the brain injury detected by magnetic resonance imaging (MRI), it is difficult to diagnose neurobehavioral disability caused by TBI in many patients. The visualization of the lesions responsible for neurobehavioral disability is essential for understanding the clinical conditions, making a diagnosis, and providing appropriate rehabilitation.

Several reports have shown that the loss of neuronal integrity could be detected using C-11 flumazenil (FMZ) positron emission tomography (PET) [4–9]. We also have reported that TBI patients had low FMZ nondisplaceable binding potential (BP_ND_) lesions indicating loss of neuronal integrity without MRI findings [4].

Similarly to FMZ PET, I-123 iomazenil (IMZ) single photon emission computed tomography (SPECT) can also be used to determine the distribution of the benzodiazepine receptor (BZR). IMZ SPECT has already been used clinically in many facilities for detecting epileptic foci in epilepsy patients.

Although IMZ is expected to show results similar to FMZ, IMZ is a SPECT tracer and FMZ is a PET tracer. Moreover, whereas the quantification method for FMZ PET has been established, quantification by IMZ SPECT is difficult and requires considerable efforts, thus limiting its use. To the best of our knowledge, there have been no studies in which FMZ PET findings and IMZ SPECT findings were compared in TBI patients, nor studies on whether those findings agree with each other.

The purpose of this study is to examine whether IMZ SPECT is as useful as FMZ PET for evaluating the loss of neuronal integrity in patients with neurobehavioral disability after TBI.

## Methods

### Patients

The subjects of this study were seven patients who suffered from neurobehavioral disability after TBI. The patients underwent FMZ PET, IMZ SPECT, and MRI.

Patient characteristics were shown in Table 1. TBI occurred at least 2 months (mean, 6 ± 5.6 months) previous to the time of FMZ PET and at least 6 months (mean, 17.7 ± 12.6 months) previous to the time of IMZ SPECT. The mean age of the patients was 30.3 years (standard deviation, 11.6 years). Of the seven patients, five were males and two were females. The causes of injury were traffic accident in five patients, assault in one patient, and sport-related in one patient. Epilepsy patients were not included in this study.

**Table 1:** Patient characteristics

Patient no.	Age (year)	Sex	WAIS-R, WAIS-III, WISC-III			RBMT	Diagnosis at the time of the accident	Period after injury (month)		Main symptoms
			FIQ	VIQ	PIQ			FMZ	IMZ	
1	28	M	98	105	90	21	No definite abnormalities	10	10	Irritability, poor concentration
2	51	M	100	99	100	20	Cerebral contusion	17	32	Difficulty of calculation
3	39	M	104	111	94	16	No definite abnormalities	4	10	Memory disorder
4	16	M	75	92	66	22	No definite abnormalities	2	13	Memory disorder
5	21	M	90	98	105	17	Cerebral contusion	2	6	Mental fatigue
6	28	F	85	84	90	23	Traumatic SAH, DAI, contusion	4	39	Memory disorder
7	29	F	106	101	101	22	ASDH	3	14	Mental fatigue

*M* male, *F* female, *WAIS-R* Wechsler Adult Intelligence Scale-Revised, *WAIS-III* Wechsler Adult Intelligence Scale-III, *WISC-III* Wechsler Intelligence Scale for Children-III, *FIQ* full intelligence quotient, *VIQ* verbal intelligence quotient, *PIQ* performance intelligence quotient, *RBMT* Rivermead Behavioral Memory Test, *SAH* subarachnoid hemorrhage, *DAI* diffuse axonal injury, *ASDH* acute subdural hematoma, *FMZ* C-11 flumazenil, *IMZ* I-123 iomazenil

### Ethics, consent, and permissions

The volunteers gave their written, informed consent in accordance with the Helsinki II declaration, and this study was approved by the Ethics Committees of Hokkaido University Graduate School of Medicine.

### PET

Images were acquired with a 5-min transmission scan and a 60-min dynamic emission scan with the HR+ PET scanner (Asahi-Siemens, Tokyo, Japan) in the 3D acquisition mode, and images were reconstructed with the brain mode of manufacturer’s software. The energy window was 350–650 keV. The acquired 3D sinograms were converted into 2D sinograms with the Fourier rebinning algorithm. The images were reconstructed by direct-inversion Fourier transformation. The reconstruction filter was a Hanning filter with 4-mm FWHM. The reconstruction matrix was 256 × 256, and the FOV was 33 cm in diameter. The full width at half-maximum (FWHM) was 6.4 mm after reconstruction. FMZ PET procedures were the same as previously described [6].

**Fig. 1:**
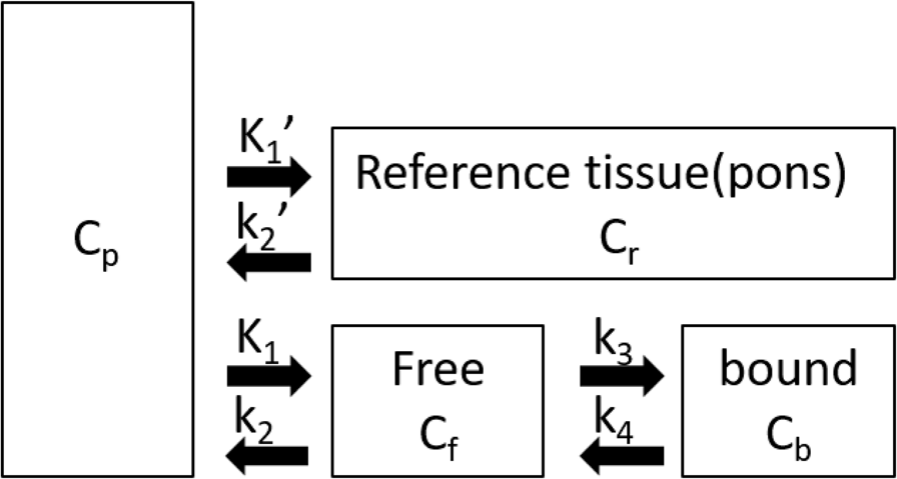
Reference tissue model, with a two-tissue compartment model for the target region and a single-tissue compartment model for the reference region

Dynamic FMZ PET was performed on all the patients. The drugs that affect BZR were withdrawn at least 1 week before FMZ PET studies. The injected dose of FMZ was 370 MBq for each patient. A set of 27 sequential PET frames of increasing duration were obtained over 60 min after FMZ injection, according to the following protocol: 40 s × 1 frame, 20 s × 10 frames, 60 s × 4 frames, 180 s × 4 frames, and 300 s × 8 frames. A reference tissue compartment model was used for a noninvasive estimation of BP_ND_ with a time-activity curve in the pons as an indirect input function (Fig. 1).

1 $$ \mathrm{d}{C}_{\mathrm{f}}/\mathrm{d} t = {K}_1{C}_{\mathrm{p}}\hbox{-}\ {k}_2{C}_{\mathrm{f}}\hbox{-}\ {k}_3{C}_{\mathrm{f}} + {k}_4{C}_{\mathrm{b}} $$

2 $$ \mathrm{d}{C}_{\mathrm{b}}/\mathrm{d} t = {k}_3{C}_{\mathrm{f}}\hbox{-}\ {k}_4{C}_{\mathrm{b}} $$

3 $$ \mathrm{d}{C}_{\mathrm{r}}/\mathrm{d} t = {K}_1\hbox{'}{C}_{\mathrm{p}}\hbox{-}\ {k}_2\hbox{'}{C}_{\mathrm{r}} $$

4 $$ {K}_1\hbox{'}/{k}_2\hbox{'} = {K}_1/{k}_2 $$

5 $$ {\mathrm{BP}}_{\mathrm{ND}} = {k}_3/{k}_4 $$

*C*_p_ is the metabolite-corrected plasma concentration (kBq/mL), *C*_r_ is the concentration in reference tissue (kBq/mL), *C*_f_ is the concentration of free (i.e., not specifically bound) ligand (kBq/mL), *C*_b_ is the concentration of specifically bound ligand (kBq/mL), *K*_1_ is the rate constant for transfer from the plasma to free compartment (mL/g/min), *k*_2_ is the rate constant for transfer from the free to the plasma compartment (/min), *k*_3_ is the rate constant for transfer from the free to the bound component (/min), *k*_4_ is the rate constant for transfer from the bound to the free compartment (/min), *K*_1_′ is the rate constant for transfer from the plasma to the reference compartment (mL/g/min), *k*_2_′ is the rate constant for transfer from the reference to the plasma compartment (/min), and *t* is time (min). The operational equation can be further simplified by assuming that the volume of distribution of the nonspecifically bound tracer in both tissues is the same as that obtained using Eq. (4). Finally, BP_ND_ was estimated by the nonlinear least method using Lammertsma’s simplified reference tissue model [10]. The parametric images were calculated with the program developed in our institute using Microsoft Visual C++ 6.0 for Windows.

### SPECT

The dose of iodine-123 IMZ was 167 MBq. SPET data were acquired from 20 to 40 min and 120 to 140 min after the tracer injection, using a triple-head gamma camera (GCA-9300/DI, TOSHIBA, Tokyo, Japan) equipped with low-energy high-resolution fan-beam collimators. The latter images were used for analysis because delayed IMZ SPECT activity and distribution volume of IMZ SPECT had high linear correlation [11]. The energy settings were 160 keV peak with 24% width. The matrix size was 128 × 128 pixels. The images were reconstructed using the filtered back-projection method without scatter correction. The data were pre-processed using a Butterworth filter with a cutoff frequency of 0.10 cycles per pixel and a power factor of eight. Attenuation correction was performed using Chang’s method. The attenuation coefficient was set at 0.10/cm. These attenuation coefficient values were determined by a phantom study. The imaging resolution was about 10 mm full width at half-maximum (FWHM) after reconstruction. Table 2 shows image acquisition and correction methods of FMZ PET and IMZ PECT study.

**Table 2:** Image acquisition and correction methods of FMZ PET and IMZ PECT study

	FMZ PET study	IMZ SPECT study
Tracers	C-11 flumazenil	I-123 iomazenil
Acquisition system	BGO PET system (Asahi-Siemens ECAT EXACT HR+)	Triple-head gamma camera with low-energy high-resolution fan-beam collimators (Toshiba GCA-9300)
Scatter correction	Single scatter simulation method	No scattter correction
Attenuation correction	Measured with transmission scan	Chang’s method
Reconstruction method	Fourier rebinning algorithm + direct-inversion Fourier transformation	Filtered back projection
Image acquisition	60-min dynamic acquisition with 3D mode	Static image (20 to 40 min and 120 to 140 min after the tracer injection)
FWHM (mm) after reconstruction	6.4	10

### MRI

MRI scan was performed using a 1.5 Tesla scanner (Magnetome Vision or Magnetome Symphony, Asahi-Siemens, Tokyo, Japan). Transaxial T2 and T2* weighted images and FLAIR images were acquired. All images were acquired with 5-mm slice thickness and no slice gap. Coronal and sagital images were added in some cases.

### Data analysis

The images obtained were assessed visually and semiquantitatively. As described previously, FMZ PET and delayed IMZ SPECT images were automatically superimposed on MRI images using multimodality image registration techniques [12].

The locations of low-uptake-level regions on FMZ PET and delayed IMZ SPECT images were visually assessed in three directions. Two specialists in nuclear medicine interpreted the images independently. When their findings did not agree, the presence or absence of low-uptake-level regions was determined by discussion.

**Fig. 2:**
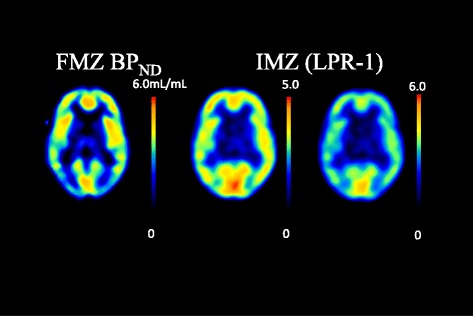
Parametric images of FMZ PET and IMZ SPECT. *Left* is FMZ BP_ND_ (0–6.0 mL/mL). *Center* image is IMZ (LPR-1) ranging from 0 to 5.0. *Right* image is IMZ (LPR-1) ranging from 0 to 6.0

A region of interest (ROI) was placed in the lesions, which was visually detected on FMZ PET images. The ROI was circle shaped of 9 mm in diameter. In delayed IMZ SPECT, a same ROI was placed in the lesion detected in FMZ BP_ND_, and the uptake of IMZ in the ROI was evaluated. The uptake of delayed IMZ was evaluated on the basis of lesion-to-pons ratio (LPR), which was corrected using the pons as the reference region. LPR corresponded to distribution volume ratio (DVR) of IMZ because delayed IMZ SPECT activity and distribution volume of IMZ SPECT had high linear correlation [11]. BP_ND_ is equal to the DVR minus a value of 1, as described by Innis et al. [13, 14]. We compared FMZ BP_ND_ and (LPR-1) of delayed IMZ SPECT (Fig. 2). The significant difference was determined by the paired *t* test and Pearson’s correlation coefficient to examine the relationship between these two parameters. A difference of *p* < 0.05 was determined as significant.

## Results

Table 1 shows the diagnosis given at the time of injury, the period after injury, the main symptoms, the scores on the Wechsler Adult Intelligence Scale (WAIS)-R [15], WAIS-III [16], or the Wechsler Intelligence Scale for Children (WISC)-III [17], and the results of the Rivermead Behavioral Memory Test (RBMT). None of the patients showed clear focal neurological dysfunction. The WAIS scores were at or below the cutoff point in two patients. Two patients had scores at or below the cutoff point in RBMT. The main symptoms were memory disorder in three patients, mental fatigue in two patients, difficulty of calculation and irritability in one patient, and irritability in one patient.

**Fig. 3:**
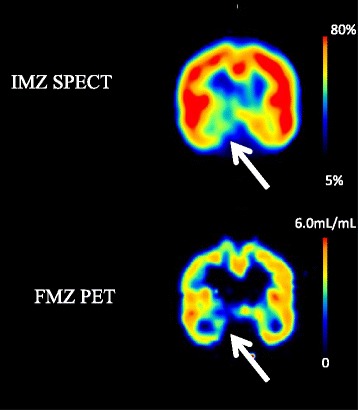
Case 1 (Patient No. 1): The patient was a 28-year-old male who was injured in a car accident 1 year previously. No abnormalities were found at the time of injury but he complained of irritability and poor concentration after the injury. Microbleeds in the right parietal lobe and left temporal lobe were observed on MRI images in the chronic phase. FMZ BP was low inside the right temporal lobe. The low uptake level inside the right temporal lobe was also observed by IMZ SPECT

**Fig. 4:**
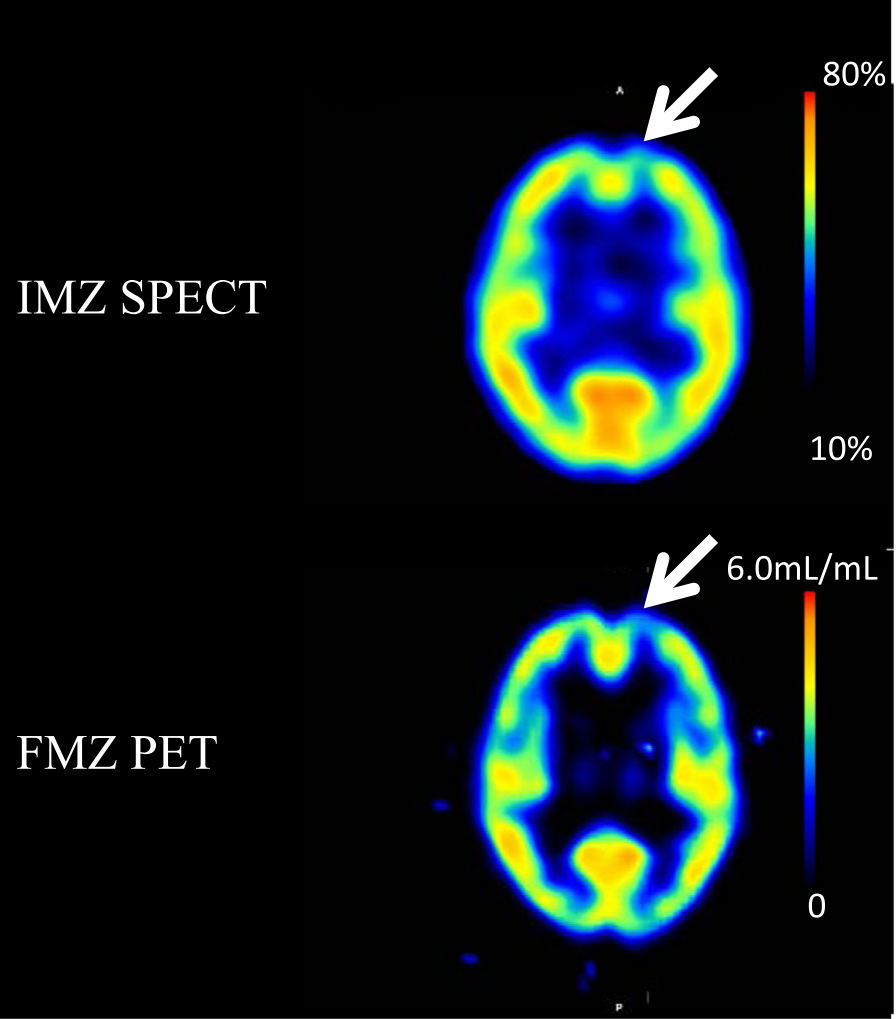
Case 2 (Patient No. 7): The patient was a 29-year-old female who was diagnosed as having acute subdural hemorrhage at the time of injury and complained of mental fatigue and headache after the injury. FMZ BP_ND_ was low inside the left frontal lobe. The low uptake level inside the left frontal lobe was also observed by IMZ SPECT

**Fig. 5:**
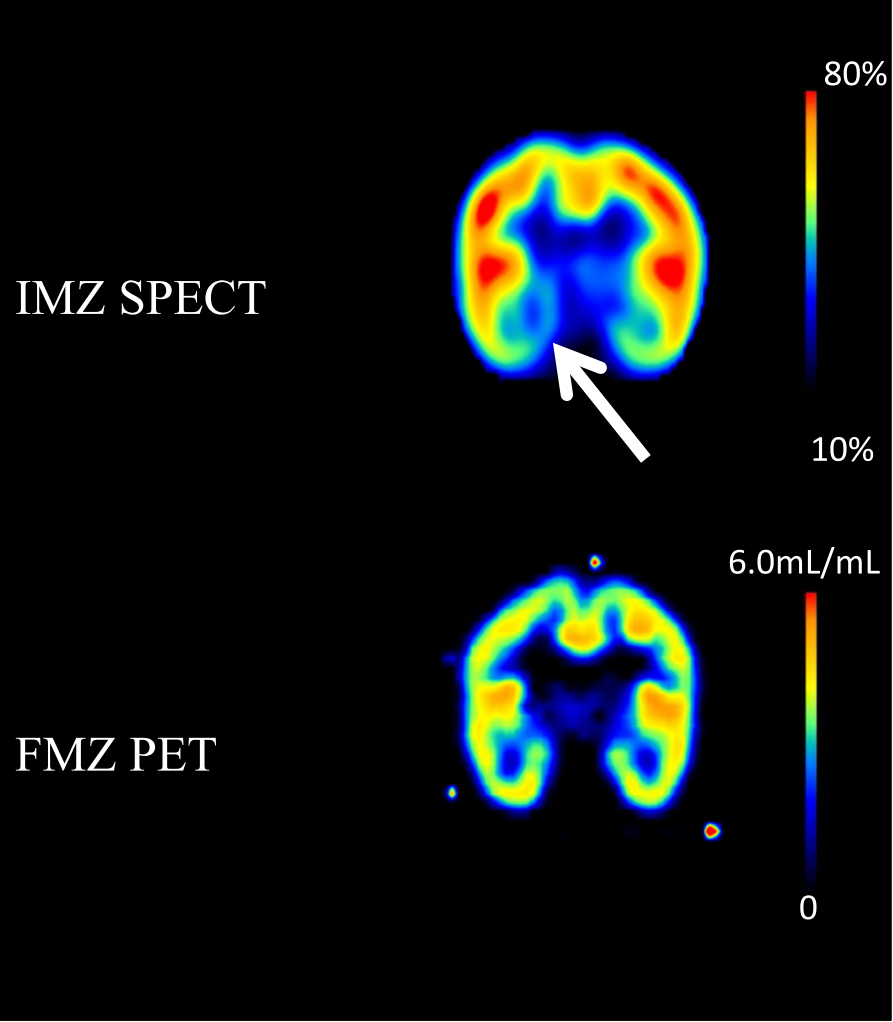
Case 3 (Patient No. 5): The patient was a 21-year-old male who was diagnosed as having right frontal brain contusion at the time of injury and complained of mental fatigue after the injury. The uptake level of IMZ SPECT was low at the right basal-medial temporal lobe. But the low BP_ND_ was not observed by FMZ PET

A decrease in FMZ BP_ND_ was visually detected in 11 regions. In delayed IMZ SPECT images, low uptakes were observed in nine lesions, eight of which also showed decreases in FMZ BP_ND_. The rate of concordance between FMZ PET and delayed IMZ SPECT was 72.7%. One lesion not detected by FMZ PET was detected by delayed IMZ SPECT (Table 3). Representative cases are shown in Figs. 3, 4, and 5.

**Table 3:** Findings in the seven patients with respect to location of low FMZ BP and location of low uptake levels on IMZ images

Patient No.	MRI		Location of low uptake on FMZ images	Location of low uptake on IMZ images
1	DAI (microbleeds)	Rt parietal lobe Lt temporal lobe	Rt medial temporal lobe	Rt medial temporal lobe
2	Contusion	Rt temporal tip Bilateral frontal lobes	Rt medial temporal lobe Lt frontal tip	Rt medial temporal lobe
3	None		Lt basal-medial temporal lobe	Lt medial temporal lobe
4	DAI (microbleeds):	Lt frontal lobe Lt parietal lobe Bilateral cerebella	Rt medial temporal lobe Lt frontal tip	Rt basal-medial temporal lobe
5	DAI (microbleeds)	Bilateral frontal lobes Bilateral temporal lobes	Lt frontoparietal lobe	Lt frontoparietal lobe Rt basal-medial temporal lobe
6	DAI (microbleeds)	Bilateral frontal lobes Bilateral internal capsules Bilateral corpus callosa	Rt basal-medial temporal lobe	Rt basal-medial temporal lobe
7	DAI (microbleeds)	Lt frontal lobe Lt temporal lobe Lt brain stem	Rt basala temporal lobe Lt frontal tip Lt frontoparietal lobe	Rt basal-medial temporal lobe Lt frontal tip

*MRI* magnetic resonance imaging, *DAI* diffuse axonal injury, *Rt* right, *Lt* left, *FMZ* C-11 flumazenil, *IMZ* I-123 iomazenil

**Fig. 6:**
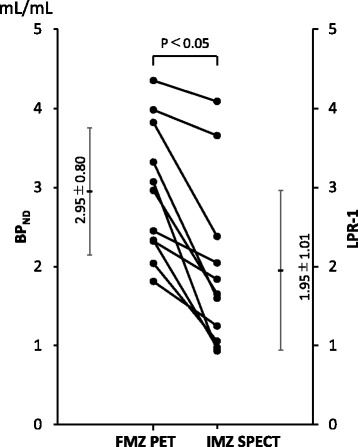
The (LPR-1) of IMZ SPECT in the 11 lesions which were detected with FMZ PET was significantly lower than FMZ BP_ND_

**Fig. 7:**
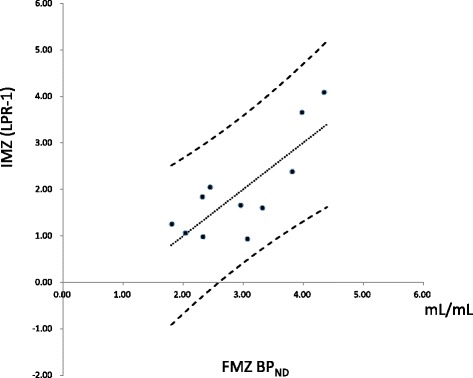
A positive correlation was found by Pearson’s correlation coefficient between the BP_ND_ in the lesions detected by FMZ PET and the (LPR-1) in the same regions obtained in IMZ SPECT (*r* ^2^ = 0.638)

The (LPR-1) of IMZ SPECT in the 11 lesions which were detected with FMZ PET was significantly lower than FMZ BP_ND_ (Fig. 6). There was significant correlation between FMZ BP_ND_ and IMZ (LPR-1) (*p* = 0.003). The correlation coefficient (*r*) between FMZ BP_ND_ and IMZ (LPR-1) was 0.80, indicating a strong positive correlation between the two (Fig. 7).

## Discussion

When FMZ BP_ND_ image was used as the reference, eight of 11 lesions were detected by IMZ SPECT. That is, most of the lesions were detected by IMZ SPECT. The sensitivity of FMZ PET in detecting lesions was slightly higher than that of IMZ SPECT. However, a lesion that was not detected by FMZ PET was detected by IMZ SPECT. IMZ (LPR-1) correlated with FMZ BP_ND_ but was significantly lower than FMZ BP_ND_.

In this study, FMZ PET and IMZ SPECT were used to identify the loss of neuronal integrity. Both FMZ and IMZ are tracers that specifically bind to central BZR. Central BZR forms a complex with the γ-aminobutyric acid type A (GABA_A_) receptor and is distributed throughout the brain cortex.

It has been reported that a low IMZ uptake level correlates with decreased neuronal density in epilepsy patients [7, 9]; and IMZ has already been used for epilepsy diagnosis in clinical practice.

In patients with ischemic cerebrovascular disorder, FMZ has been reported to be a useful marker indicating the presence of irreversible changes in neurons, even in a region where abnormalities were not found by MRI [6, 8].

Several reports have shown that the loss of neuronal integrity in TBI patients can be evaluated by FMZ PET [4, 5]. We have reported that even in patients with head injury showing no abnormal MRI findings, regions with a decreased cerebral metabolic rate of oxygen (CMRO_2_) were identified by ^15^O-labeled gas PET, and that a decrease in BP_ND_ was observed on FMZ PET images in some of those regions. The decrease in FMZ BP_ND_ may be related to the loss of neuronal integrity, and it seemed that the decrease in CMRO_2_ in the regions showing no loss of neuronal integrity was due to functional hypometabolism [4]. Moreover, in the FMZ PET of patients with diffuse axonal injury (DAI), abnormalities were observed in regions showing no abnormal MRI findings [5].

FMZ PET and IMZ SPECT are considered to be useful for identifying the loss of neuronal integrity. However, a comparison between FMZ PET and IMZ SPECT in epilepsy patients showed that FMZ PET more accurately detects epileptic foci, whereas the accuracy of IMZ SPECT was lower [18]. There has been no report that FMZ PET findings and IMZ SPECT findings agree with each other in TBI patients. In this study, most of the IMZ SPECT findings agreed with the FMZ PET findings, suggesting that IMZ SPECT was as useful as FMZ PET for evaluating the loss of neuronal integrity in TBI patients. However, it should be noted that the IMZ SPECT and FMZ PET findings were not completely identical because of the inherent differences between the tracers (FMZ and IMZ) and image generation methods; PET v.s. SPECT.

### Inherent differences between PET and SPECT and their tracers

The direction of gamma rays is determined by a collimator in SPECT. PET can determine photon origin using coincidence detection, it does not require a collimator. A collimator attenuates large number of incoming radiation; therefore, PET has an increase in sensitivity of two to three orders of magnitude compared to PET. PET has excellent performance in resolution and sensitivity [19]. Moreover, PET is superior to SPECT in quantitative performance because absorption is directly measured in PET, or calculated by CT in PET/CT, whereas that in SPECT is only corrected using a mathematical model (Chang’s method). FMZ PET, which has a high spatial resolution, is more accurate than IMZ SPECT because abnormalities are determined on the basis of the visual assessment of low-uptake-level regions. To address the issue of spatial resolution, a high sensitivity SPECT system was used in this study. This may be the reason why the accuracy of IMZ SPECT in this study was higher than that of the report on the comparison of FMZ PET and IMZ SPECT in epilepsy patients [18].

FMZ is an antagonist of BZR [20]. In the brain, 80–90% of FMZ specifically binds to BZR and the rest exists as free or nonspecifically bound FMZ [21–23]. Moreover, the quantification method for FMZ PET has been established. FMZ is taken up by the brain immediately after its injection and is then promptly washed out. Quantitative values can be obtained by 1-h dynamic scanning immediately after the injection of FMZ. IMZ is a partial inverse agonist. The BZR affinity of IMZ is 10-fold that of FMZ, and the specific-to-nonspecific binding ratios are 40–50:1 [24, 25]. The quantification method for IMZ SPECT has not been established. FMZ PET BP_ND_ images are quantitative images that can exclude nonspecific accumulation. On the other hand, IMZ SPECT images are qualitative images that cannot exclude nonspecific accumulation, leading to decreased contrast and decreased sensitivity for detecting abnormalities. In contrast, IMZ is an agonist, unlike FMZ, having pharmacological effects. IMZ is designed as a tracer that has low ligand occupation rate because of pharmacological effect.

The accumulation of LPR in IMZ SPECT was determined in this study. The reference tissue for FMZ BP_ND_ is the pons, which shows little specific accumulation of BZR. Therefore, the accumulation LPR as determined by IMZ SPECT was compared with the quantitative value obtained by FMZ PET. This may be the reason why the semiquantitative accuracy of IMZ SPECT was improved and the IMZ (LPR-1) showed a high level of correlation with FMZ BP_ND_.

### Decrease in expression level of BZR

There are three possible causes of a decrease in BZR expression level. The first is direct injury. Brain contusion is often located at the bottom, outer surface, and inner surface of the frontal and temporal lobes, or in the temporal pole [26]. In this study, all the regions showing a decrease in BZR expression level were located in the frontal and temporal lobes, with most of them located at the bottom, inside, and in the pole of these lobes. The injuries seemed to have been caused by the bone of the skull base.

The second is DAI. DAI is an axonal injury due to the shear or distortion induced by rotational acceleration and is often located at the corticomedullary junction or in deep white matter [27, 28]. It has been reported that an axonal injury slowly leads to Wallerian degeneration and results in delayed neuronal death [29]. Although BZR is not suitable for detecting disorders in the white matter because it exists in the gray matter, the abnormalities detected may indicate neuronal death caused by DAI. In this study, five patients had DAI identified by MRI.

The third is apoptosis. Apoptosis was observed in the injured brain cortex and white matter in a study using mild TBI models, indicating that TBI was a cause of cell death [30].

In this study, there was a region where abnormalities were detected only by IMZ SPECT. Such abnormalities may be a false positive resulting from the limited resolution of SPECT and the issues of accuracy of the test method such as the lack of quantification. Moreover, the results may be affected by the difference in the time point at which FMZ PET and IMZ SPECT were carried out.

In this study, IMZ SPECT was carried out several months after FMZ PET. Therefore, there was a possibility that secondary changes due to TBI, including Wallerian degeneration and apoptosis, led to the decrease in BZR expression level, and, as a result, the abnormalities not detected by FMZ PET were detected by IMZ SPECT. Further research is required to clarify the temporal changes detected by IMZ SPECT.

### Correlation of FMZ BP_ND_ and (LNR-1) of IMZ

We compared FMZ BP_ND_ and (LPR-1) of delayed IMZ SPECT. In previous study, BP_ND_ of IMZ SPECT were much higher than (LPR-1). If IMZ binds about 10 times as strong as FMZ, (LPR-1) will be 10 times larger than BP_ND_ of FMZ. Millet et.al reported that BP_ND_ of IMZ was about 5 times that of FMZ. Considering that the spatial resolution of SPECT is worse than that of PET, BP_ND_ of IMZ is considered to be higher than that of FMZ by more than 5 times. One reason was considered to be derived from scatter correction. We did not correct the scatter in SPECT study. This SPECT system is equipped with Triple energy windows (TEW) scatter correction. Because the TEW method was reported to increase the noise [31] and visual interpretation was difficult in some cases, we decided to analyze SPECT images without scatter correction. Scatter correction is important in the quantitative analysis of brain SPECT. Skull base has high attenuation [32] and therefore generates a lot of scatter photons. Axelsson et al. [33] reported that the relation between true and measured concentration ratios was almost linear after scatter correction and the effect of scatter correction was larger in the low counts area than in the high counts area. The activity of pons might be overestimated in this study. Other reason was derived from static data acquisition. We did not perform dynamic study in SPECT. Dynamic study was necessary to calculate accurate DV and BP in both PET and SPECT study. Other reason was derived from attenuation correction. Absorption correction of PET is based on mu map directly measured in PET, or calculated by CT in PET/CT, and it was more accurate than that of SPECT system.

### Clinical significance of IMZ SPECT

Cerebral blood flow SPECT is more sensitive to TBI-associated dysfunction than MRI and computed tomography (CT) [34, 35]. However, blood flow and glucose metabolism change in connection with the activities of the patient examined and therefore reflect even a functional decline that is not due to a neuronal abnormality. The neuron loss can be more accurately detected using BZR-specific ligands.

It is considered that FMZ PET has higher diagnostic accuracy than IMZ SPECT. However, ^11^C-FMZ must be purified in a hospital because its half-life is as short as 20 min. It can be used only for research purposes and is difficult to use in clinical settings. On the other hand, IMZ has long radioactive half-life, it is easy to handle it. And it can be used in many facilities because it is more easily available than FMZ.

Because IMZ SPECT is superior to FMZ PET in terms of versatility, it is preferable to use IMZ SPECT instead of FMZ PET to detect the loss of neuronal integrity.

The detection of the loss of neuronal integrity using IMZ SPECT enables the evaluation of the distribution of lesions that cannot be observed by MRI. IMZ SPECT will contribute to the understanding of the cause of neurobehavioral disability in patients with head injury but showing no abnormalities on MRI images. The clinical symptoms can be better understood when the site of lesions is identified, leading to the improvement of insights into the disease and the promotion of the understanding of affected patients by their families and surrounding persons. Moreover, although presently the treatment of neurobehavioral disability mainly involves supportive care, such as the development of compensatory approach through rehabilitation, the identification of the site of lesions will lead to a curative treatment with the development of regenerative medicine in the future.

### Limitations

The number of patients involved in this study was small. Further research with a larger number of patients is required in the future.

As for PET system, we used Siemens HR+ PET system for FMZ PET study. HR+ PET system was a high spatial resolution BGO PET system (4.39 mm@1 cm in transaxial: NEMA NU-2001) and with relatively high sensitivity (6.65kps/MBq@350KeV NEMA NU-2001). We think that this system was still good only for brain study; however, it seemed to be old system in these days. It had no CT system and lower energy resolution (23%) than LSO or LYSO system (10–14%). State-of-the-art time-of-flight PET/CT system is better for further analysis.

As for SPECT system, we used triple-head gamma camera. Triple-head gamma camera with fan-beam collimator had high spatial resolution with high sensitivity. Our results might be attributed to these features of triple-head gamma camera. As two-head gamma cameras are prevalent these days, the results might not be universal.

We did not correct scatter in SPECT study. New scatter corrections without increasing noise were developed [11]. With those scatter corrections, the correlation of semiquantitative parameters of PET and SPECT might become better without affecting visual diagnosis.

## Conclusions

IMZ SPECT is as useful as FMZ PET for evaluating the loss of neuronal integrity in patients presenting with neurobehavioral disability after TBI.
